# Treatment of Painless Nodules With Glucopuncture in Dupuytren's Disease in Men: A Clinical Case

**DOI:** 10.7759/cureus.31445

**Published:** 2022-11-13

**Authors:** Jan Kersschot, Thomas Mathieu

**Affiliations:** 1 Family Medicine, Private Practice, Antwerp, BEL; 2 Physical Medicine and Rehabilitation, Antwerp University Hospital, Antwerp, BEL

**Keywords:** glucopuncture, painless nodule, dextrose prolotherapy, isotonic sugar water, local injection, growth factors, palmar fascia, fibromatosis, case report, dupuytren's disease

## Abstract

Since there are currently no interesting treatment options for men with early-stage Dupuytren's disease, new, safe and effective treatment methods are required to improve the quality of life of such patients. Over the past decade, isotonic sugar water injections have received increasing attention from clinicians worldwide. Glucopuncture is a new term to describe isotonic sugar water injections into dermis, fascia, muscles, tendons and ligaments. In this clinical case, a 75-year-old man was treated with glucopuncture for a painless nodule on the right hand. A marked reduction in hardness and size of the nodule was observed after six sessions. Establishing a case series with imaging before and after treatment could be a first step to further illustrate the effects of this novel injection technique. Next, randomized controlled trials (RCTs) with sufficient sample size are needed to establish the value of glucopuncture for painless nodules in Dupuytren's disease.

## Introduction

Dupuytren's disease (DD) is a common fibromatosis of the palmar and digital fascia [1,2]. The palmar aponeurosis of the hand is a fascia composed of collagen that plays a key role in the ability to grasp and hold objects. DD begins in the palm as nodules that slowly form along longitudinal tension lines. DD is a connective tissue disorder that can evolve over time into progressive contracture of the fingers [2,3]. The most commonly affected fingers are the fourth and fifth. Over months or years, such contractures can lead to deformities in the palmar aponeurosis that can severely impair the quality of life [3,4].

The overall prevalence of DD is 0.2% [5]. It is about five times more common in men than in women [6]. A genetic and ethnic predisposition has been identified. Smoking, diabetes, epilepsy, hypercholesterolemia, manual labor (e.g., screwdriving) and exposure to vibration (e.g., jackhammer) are possible risk factors [5-7].

Fibrotic disorders such as DD are characterized by abnormal fibroblast proliferation and matrix deposition in the palmar fascia [8,9]. An inflammatory infiltration of palmar tissue is described [8]. Numerous cytokines are involved, such as interleukin-1 and connective tissue growth factor [9,10]. The pathophysiology of DD involves abnormal fibroblast growth in the fascia, which consists mainly of type III collagen. Transforming growth factor-β1, interleukin-1β and vascular endothelial growth factor are considered potential therapeutic targets for the treatment of DD [9]. Recent studies illustrate the importance of STAT1-modulated IL-13Rα1 response [10].

DD usually starts as a palmar nodule. This nodule may gradually develop into a hard chord in the palmar fascia which limits the extension of the affected finger progressively over time [1-3]. Collagen-rich cords gradually progress into permanent contracture deformities which feel like hard painless strings [3,4]. Although physical examination gives at most a *subjective *idea of the extent of DD, it is still the gold standard for assessing disease stage and progression [11].

Standard treatment protocols to treat Dupuytren's contracture are open fasciectomy, needle aponeurotomy and enzymatic fasciectomy [12-14]. Injections with collagenase clostridium histolyticum may be satisfactory in some patients [12,15]. In this clinical case, isotonic sugar water (ISW) injections such as glucose 5% or dextrose 5% are presented as a new treatment modality to reduce the size and hardness of palmar nodules. The injection procedure itself is easy and safe. To make the injections into the palm of the hand less painful, the patient could apply a topical anesthetic agent an hour before the procedure. As the total amount of glucose injected each session is very small, these injections can be applied to patients with diabetes.

## Case presentation

**Figure 1:**
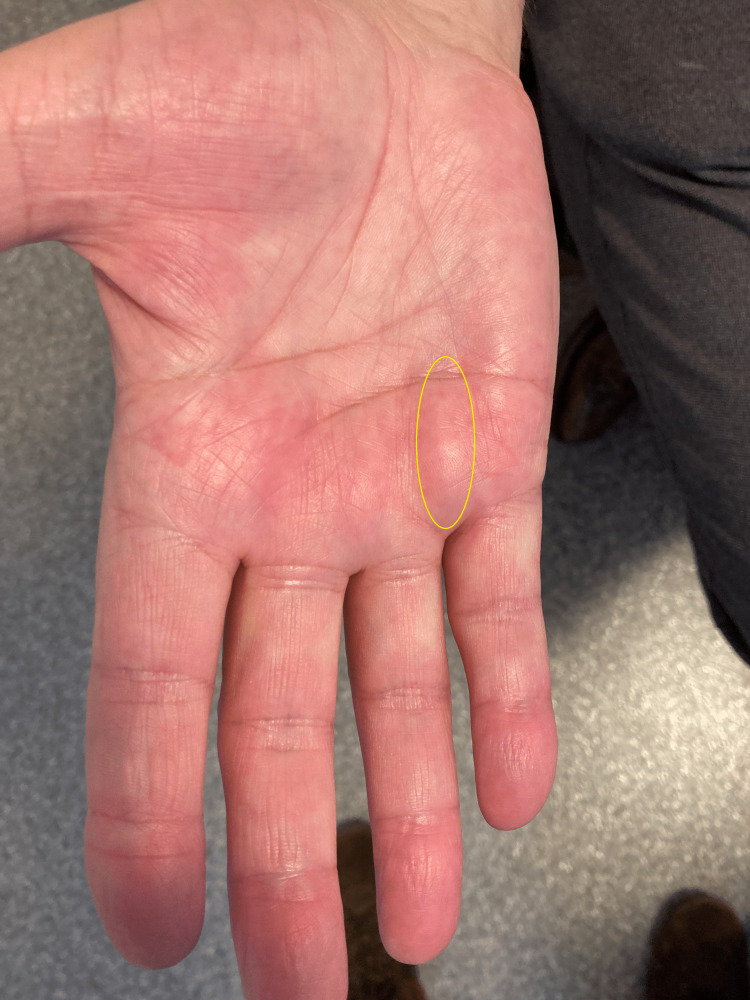
Nodule in the Right Hand

This case report describes the history and treatment course of a 75-year-old retired businessman (born Oct. 20, 1947) who presented with a painless, palpable, firm nodule in his right hand that had been present for three months. At the first visit (Feb. 21, 2022), a nodule approximately 2.5 cm (about 1 in) by 0.5 cm (about 0.2 in) was found in the palm of his hand, located between metacarpals four and five (Figure 1). At the beginning of treatment, the patient did not mention any other clinical signs except a mild discomfort while shaking hands. No ultrasound or MRI was performed before treatment as it was not planned to use this case for publication in a medical journal. The hardness of the lump was assessed on a 0 to 10 scale, with 0/10 representing normal tissue and 10/10 representing a very hard string (as found in grade 3). At the first visit, the hardness was rated as 6/10 (the same hardness as the tendon of the extensor pollicis longus in the anatomical snuffbox). The nodule was not tender on palpation. The Hueston's tabletop test (HTT) was positive (angle of 30 degrees above the horizontal plane, measured on the ulnar side).

**Figure 2:**
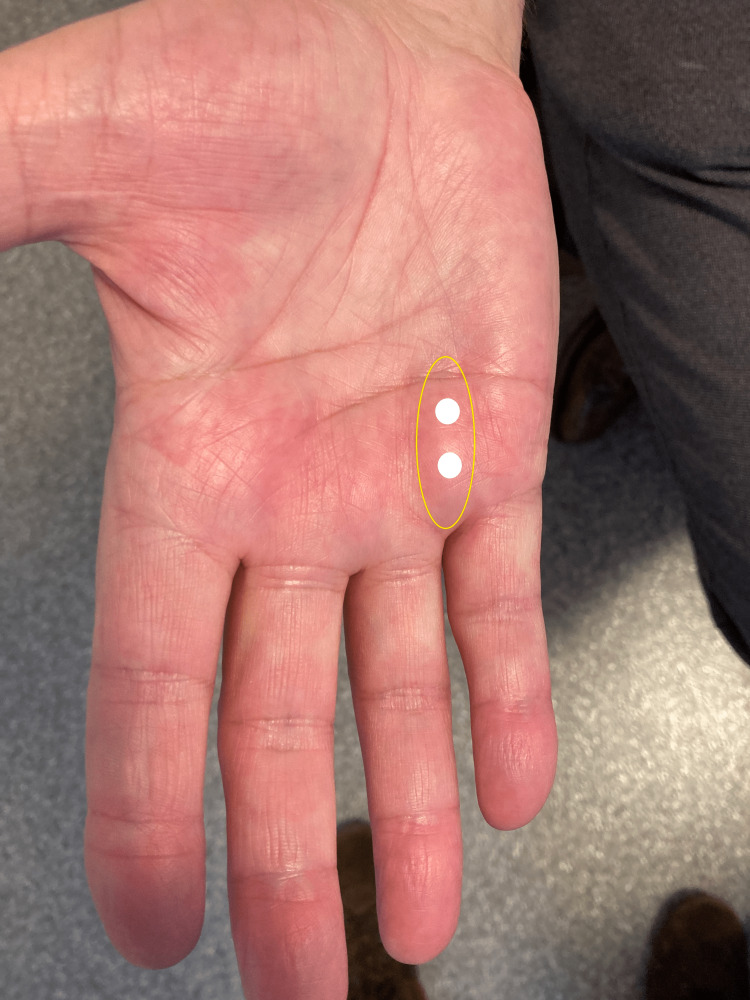
Two Injections into the Right Hand

After disinfection of the skin, a 2mL syringe with a 30G needle was used to administer the injections. The patient did not apply a topical anesthetic before the procedure. He received glucose 5% injections at two sites in the nodule (2 x 1 mL) (Figure 2). No local anesthetics nor steroids were added to the sugar water. The injection depth was only a few mm (about 0.1 in). The injections were given as close to the nodule as possible. Injecting straight into the nodule itself was very difficult because the fibrotic tissue was very hard. After the first session, a moderate improvement in the hardness of the nodule was found (5/10). However, the size of the nodule was still the same (2.5 cm x 0.5 cm). HTT also remained the same (30 degrees above the horizontal plane).

On the second and third visits (March 9 and 16), he received the same injections as before (2 x 1 mL, 30G needle). After the third session, there was a significant reduction in nodule hardness (3/10) and size (2 cm x 0.3 cm). The Hueston's tabletop test (HTT) was now 15 degrees above the horizontal plane. At the fourth and fifth visits (March 24, April 14), he again received 5% glucose injections into the nodule. He received a total of 5 mL (2 x 2.5 mL) with a 27G needle. Using a thicker needle is easier to inject the solution into fibrous tissue, but it is also more painful. After the fifth session, the size was 1.5 cm x 0.2 cm, HTT negative (0 degrees) and hardness 1/10. On the sixth visit (April 27), he received three injections (total volume about 5 mL) with a 27G needle (see Video 1). He received approximately 3 mL, 1.5 mL and 0.5 mL at three different sites in and around the nodule.

**Video 1:** Three Injections of Glucose 5% into the Right Hand (5 mL)

**Figure 3:**
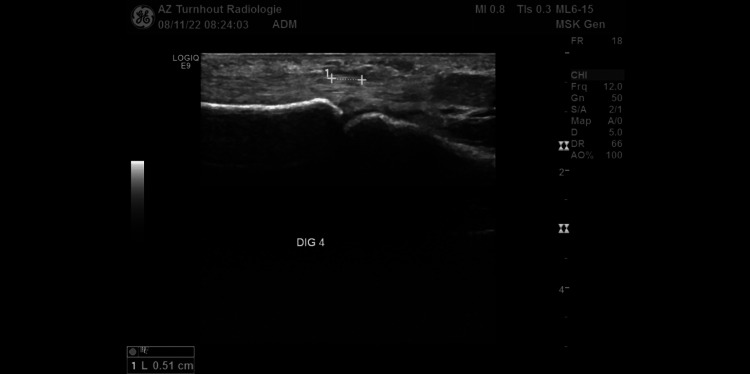
Ultrasound Investigation Flexor Digit 4 (Lesion 0.51 cm)

After the sixth session, there was an additional reduction in nodule hardness (0/10). It was now impossible to identify the nodule clinically. The patient said there was no longer discomfort when shaking hands. No further treatment was needed at the seventh visit (May 11). Clinical examination showed no palmar nodule. The HTT was still negative (0 degrees). Several months later, the nodule had not recurred (checked by e-mail: August 2022). The patient did not attend a physical therapy program. An ultrasound examination on November 8, 2022 showed no nodular fibrotic lesions in the palmar fascia. A lesion of 5.1 mm (0.2 in) was found on the flexor of digit 4 (Figure 3) and a lesion of 9.7 mm (0.4 in) was found on the flexor of digit 5. A follow-up in six months was planned.

## Discussion

This article hypothesizes that local injections of isotonic sugar water (ISW), such as glucose 5% in water (G5W) or dextrose 5% in water (D5W), can be used to treat painless nodules in men with DD. Glucopuncture (GP) is a new term for palpation-guided or landmark-guided ISW injections [16]. Usually, these ISW injections are performed on an outpatient basis *without *ultrasound or fluoroscopy guidance. Since sugar water is available worldwide and inexpensive, the procedure is interesting for medically underserved populations [17]. GP differs from prolotherapy (PT) because PT typically uses *hypertonic *sugar water (HSW) injections (glucose or dextrose 10-20%) that evoke osmotic cell death, followed by an inflammatory response, subsequent proliferation (hence the term prolotherapy) and tissue repair [18]. HSW solutions are always mixed with a local anesthetic (LA). In GP, shallow injections are typically used into soft tissues such as dermis, fascia, muscles, tendons, entheses and ligaments [19,20] whereas in PT, injections are mainly given into ligaments, tendons, entheses and joint cavities (Table 1).

**Table 1:** Difference between GP (Glucopuncture) and PT (Prolotherapy)

	GP	PT
Isotonic Sugar Water (ISW)	x	
Hypertonic Sugar Water (HSW)		x
Local Anesthetics (LA)		x
Dermis	x	
Fascia	x	
Muscle	x	
Tendon	x	x
Enthesis	x	x
Ligament	x	x
Joint		x

The mechanism of action (MOA) of sugar water injections is still under debate. It is hypothesized that ISW injections can support cell metabolism through ATP [19, 20]. Substance P, transforming growth factor-β1, vascular endothelial growth factor and interleukin-1β are considered potential therapeutic targets in the treatment of DD [8,9], but it remains unclear if and how these ISW injections interfere with these pathways. It may also be worth investigating the possible effects of ISW injections on the STAT1-modulated IL-13Rα1 response [10]. Clearly, more research in this area is required to establish a solid MOA for GP.

## Conclusions

This case report aims to convey to the medical community why this particular observation is relevant in the context of current knowledge about the treatment of early stages of Dupuytren's disease in men. Several clinicians worldwide have found glucopuncture to be an inexpensive, safe and easy-to-apply treatment option for non-rheumatic musculoskeletal disorders. In this clinical case, clinical improvement was observed after six sessions of glucopuncture. An ultrasound investigation six months after the last session showed only two minor lesions on the flexor tendons of digit 4 and 5. Areas of weakness in this case are the absence of standardization of the injection method and the lack of radiological follow-up before and during treatment. It would be interesting to assess the evolution of the lesions with ultrasound or MRI in a large case series.
